# Phenolic Acids Commonly Found in Natural Products Modulate Protein Aggregation in *Caenorhabditis elegans* Neurodegeneration Models

**DOI:** 10.1021/acsomega.5c00695

**Published:** 2025-06-26

**Authors:** Xareni Valle-Jiménez, Ixchel Osorio-Paz, Silvestre Alavez

**Affiliations:** 414538 Universidad Autonoma Metropolitana - Lerma, Lerma, State of México 52005, México

## Abstract

Abnormal protein accumulation is frequently associated with the gradual degeneration of the central nervous system, which results in the development and progression of several neurodegenerative diseases (NDs). Since the incidence of ND is on the rise, their effects represent a substantial psychological and economic burden. As we advance in understanding human aging mechanisms, it is desirable to accelerate the discovery of molecules that can modulate human aging and perhaps postpone the onset of age-related disease. Therefore, uncovering compounds that can prevent the formation of protein aggregates should be a priority in the aging research field. Phenolic acids are organic compounds found in many natural products, such as vegetables and fruits. These compounds have been shown to have potential neuroprotective benefits. However, its effects on protein aggregation related to neurodegeneration processes are still not clear. In this study, we thoroughly explored the ability of four phenolic acids: caffeic (CA), p-coumaric (p-CoA), ferulic (FA), and gallic (GA) acids to prevent protein aggregation in three *Caenorhabditis elegans* models of human neurodegeneration, such as Alzheimer’s disease, Huntington’s disease, and Parkinson’s disease. We found that high CA, p-CoA, FA, and GA concentrations reduce the β-amyloid-aggregation-induced paralysis phenotype by up to 32%. Also, high CA, FA, and GA concentrations decreased paralysis percentage and polyQ aggregations by 25, 26, and 47%, respectively. Interestingly, high concentrations of p-CoA reduced polyQ aggregation but not the percentage of protein aggregation-induced paralysis. Additionally, only high concentrations of CA, along with lower concentrations of FA and GA, demonstrated the potential to reduce α-synuclein aggregation. Our findings suggest that CA, FA, and GA are worthy candidates for acting as neuroprotectors in mammals.

## Graphical abstract

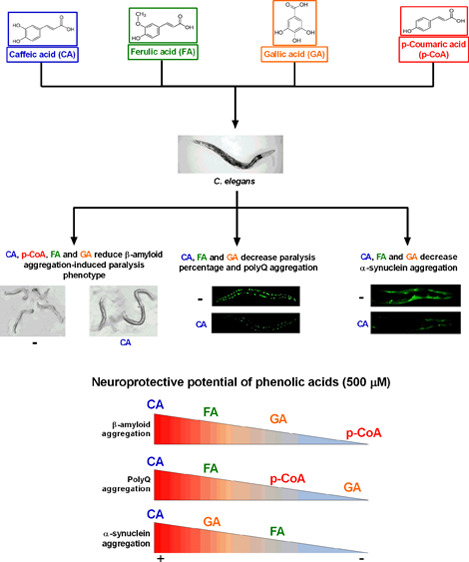

## Introduction

Aging is a physiological process closely associated with the onset and progression of neurodegenerative diseases (NDs). NDs are a heterogeneous group of diseases that affect the central nervous system and are characterized by progressive neuronal loss in specific brain areas, probably due to the accumulation of protein aggregates. These diseases are not yet curable and present cognitive, motor, functional, emotional, economic, and social consequences, making them a growing burden to society.^1,2^ According to the World Health Organization, as long as preventative therapies and viable treatments remain elusive, the increasing prevalence of NDs is one of the most significant public health challenges nowadays and in the decades to come.^3^

Individuals with ND present multiple clinical complications, which can lead to constraints on their quality of life. These disorders can affect various systems in the body, including the motor system, leading to conditions such as ataxias, as well as the cognitive system, which can cause dementias.^4^ The molecular mechanisms underlying ND are unclear and involve genetic and environmental factors. The exact causes of ND are currently unclear due to the interplay of these factors. Current research in genetic epidemiology suggests that specific genes encoding for proteins prone to oligomerization and aggregation (i.e., α-synuclein and amyloid precursor protein)^5^ or those involved in autophagy and mitochondrial metabolic stress (i.e., PINK1, Parkin)^6^ may contribute to the development of NDs. Therefore, it is essential to uncover compounds that can prevent the formation of protein aggregates or modulate autophagy to address the issues caused by their accumulation.^7,8^ Ideally, such compounds should be innocuous, easy to administer, and accessible to the general population.

Phenolic acids are nonflavonoid phenolic compounds widely present in vegetables, fruits, and beverages such as green tea, coffee, red wine, and beer. Including these products in the daily diet can positively impact overall health.^9–11^ Many reports suggest that this beneficial effect is due to its antioxidant capacity, which is conferred by the scavenging activity of a hydroxyl group in its ring.^12^ However, abundant research has also explored the intracellular molecular pathways activated by phenolic acids. In *C. elegans*, for example, it has been shown that some phenolic acids can decrease the aggregation of several molecules associated with the development of NDs, enhance autophagy activity via helix loop helix-30 (HLH-30),^13^ improve protein homeostasis,^14^ or protect against toxicity via abnormal Dauer formation-16 (DAF-16).^15^

After reviewing the existing literature, we selected four structurally similar phenolic acids that are commonly found in many natural products, making them easily accessible for incorporation into the human diet: caffeic acid (CA), p-coumaric acid (p-CoA), ferulic acid (FA), and gallic acid (GA). In humans following a balanced diet, phenolic acids are present in a free form in the bloodstream, where p-CoA represents approximately 70%, while CA and FA account for 10–20% of the total.^16^ These compounds have shown different beneficial effects on health, including anti-inflammatory, antidepressant, and neuroprotective effects,^17^ such as the inhibition of the LPS-induced microglial inflammation.^18^ However, its role in halting protein aggregation related to NDs is still being explored.

The nematode *C. elegans* is very well suited to whole-organism screens because it combines the ease of cell culture with the complexity of a multicellular nonvertebrate organism. *C. elegans* has a short lifecycle, a brief reproduction time, and shares many genetic and cellular similarities with humans.^19,20^ Neurobiology studies frequently use this nematode since a wholly mapped neural network and the connectome have already been described.^21^ Several models of human neuropathologies have been developed in this nematode, including those for Alzheimer’s disease (AD), Huntington’s disease (HD), and Parkinson’s disease (PD), where human β-amyloid peptide, polyglutamine (polyQ), and α-synuclein, respectively, accumulate in the muscles.^22^ Therefore, by focusing on the accumulation and aggregation of specific proteins, this biological model can uncover new therapeutic compounds that can delay the progression of these NDs.

It is important to note that most studies searching for geroprotectors typically focus on a limited range of concentrations. In a previous study, we thoroughly tested a broad range of concentrations and found a striking effect of vanillic acid in *C. elegans*, more significant than the previously reported.^14^ Therefore, this study explores the effect of a wide range of CA, p-CoA, FA, and GA concentrations on protein aggregation-induced paralysis in three well-studied *C. elegans* models of neurodegeneration: AD, HD, and PD.

## Results and Discussion

Recognizing that comprehensive dose–response experiments can uncover concentrations that improve or enhance previously unreported beneficial biological effects, we performed thermotolerance assays over a wide range of concentrations (0.1 to 2500 μM). Figure 1 displays the complete results of the dose–response experiments, including those that do not cause thermotolerance in N2 worms at 35 °C. Results show that the treatment with 500 and 2500 μM CA increased survival by 20.7 ± 2.7 and 16.1 ± 0.38%, respectively; 100, 300, and 750 μM p-CoA in 21 ± 1.14, 15.7 ± 0.75, and 14 ± 0.71%; 0.1 μM FA in 13 ± 1.9%; and finally, 500 μM GA in 19.4 ± 1.34% (Figure 2). Therefore, follow-up experiments were conducted at similar doses.

**Figure 1:**
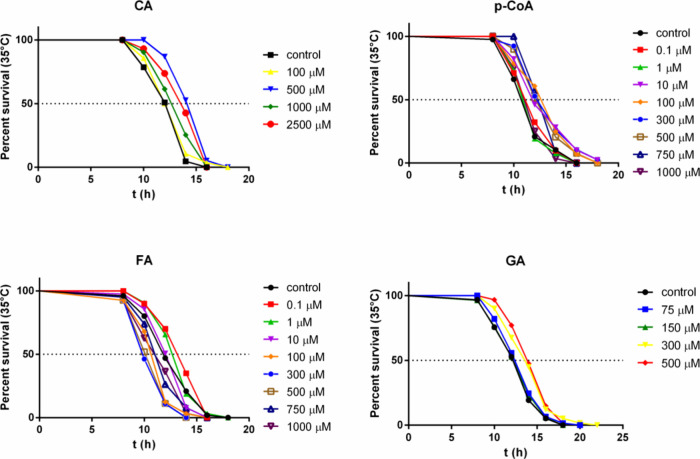
Dose–response curves of phenolic acids effect on*C. elegan’s* thermotolerance. Kaplan–Meier survival curves of N2 worms treated with different concentrations of Caffeic (CA), p-Coumaric (p-CoA), ferulic (FA), and gallic (GA) acid or vehicle as control at 35 °C.

**Figure 2:**
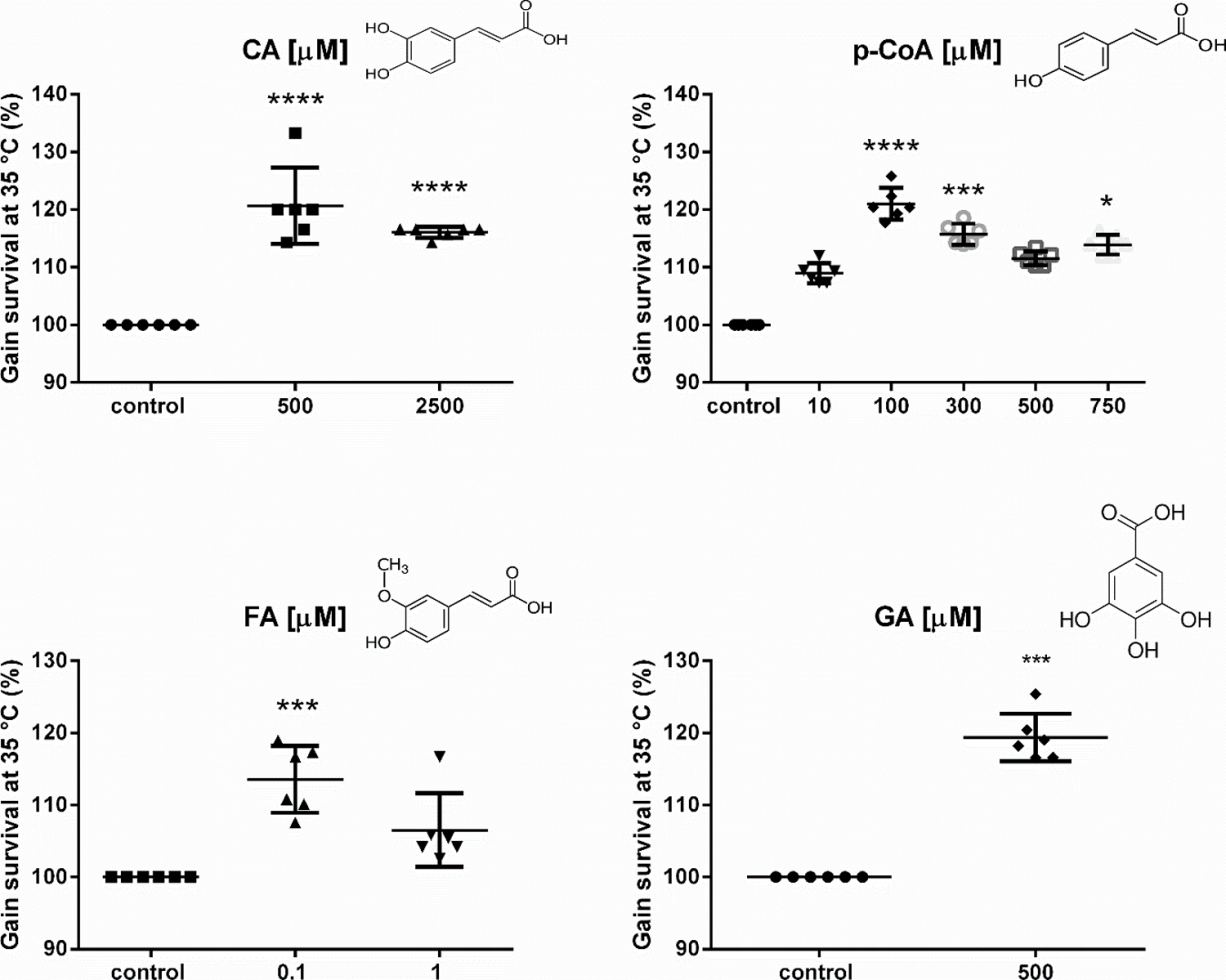
Effect of phenolic acids on thermotolerance in*C. elegans*. N2 worms were cultured at 35 °C under different concentrations (μM) of Caffeic (CA), p-Coumaric (p-CoA), ferulic (FA), and gallic (GA) acids and control. The graphs show the gain survival percentage of six independent experiments with SD error bars. Statistical significance was determined by the Kruskal–Wallis test and Dunnet’s multiple comparisons * *p* < 0.05, *** *p* < 0.001, ****p < 0.0001.

Since resistance to thermal stress in *C. elegans* is related to longevity,^23^ evaluating the thermotolerance of N2 worms exposed to different concentrations of phenolic compounds is a valuable tool to approximate their potential as neuroprotectors. Our results show that high concentrations (100–2500 μM) of CA, p-CoA, and GA increase thermotolerance by approximately 14–21%. On the other hand, only very low concentrations (0.1 μM) of FA showed a significant increase of around 14%. These results show that phenolic acids, despite their structural similarity, have a different effect on cells, and this could be due to their bioavailability. The concept of bioavailability incorporates bioaccessibility, absorption, tissue distribution, and bioactivity.

Different factors can influence the bioavailability of a compound; the first factor is the bioaccessibility or absorption in the gastrointestinal tract.^24^ Absorption of FA in the intestine probably occurs by passive diffusion. However, the possibility of a saturable FA transport via a monocarboxylic acid transporter dependent on pH has also been reported.^25^ These results suggest that the nematode could absorb FA more efficiently and in greater quantity than the other phenolic acids due to its methoxy group. Consequently, this increase in absorption could modulate the activation of the molecular mechanisms. In a recent work, it has been shown that higher concentrations of FA (500 μM) improve longevity and stress resistance by modulating antioxidant and autophagy genes such as *sod-3*, *gst-4*, *lgg-1,* and *hsp-16.2*.^26^ However, it is unknown whether lower concentrations could have the same effect.

Since we aim to explore the effect of phenolic compounds on protein aggregation associated with NDs and aging, we perform paralysis assays with well-studied *C. elegans* models of neurodegeneration. In this work, we focused on three *C. elegans* models of NDs associated with protein aggregation: AD, HD, and PD.

The presence of β-amyloid protein plaques and neurofibrillary tangles of hyperphosphorylated tau protein characterizes AD. Both of these proteins accumulate in the brain several years before the manifestation of clinical symptoms.^27^ To determine the impact on β-amyloid aggregation, we used the CL4176 (*smg-1ts [myo-3/A*β*1–42 long 3′-UTR]*) strain. CL4176 grows normally at 15 °C, but when the temperature is raised to 25 °C, this strain expresses the human β-amyloid_3–42_ peptide, driven by the *myo-3* promoter, producing β-amyloid protein aggregates in the muscles. This protein aggregation induces a characteristic paralysis phenotype, where the worms display a straight position, lack of movement, and a halo of digested bacteria around the head (Figure 3B). After 36 h of treatment with 2500 μM CA, 750 μM p-CoA, 1000 μM FA, and 500 μM GA, this paralysis phenotype is reduced by approximately 29 ± 2.2, 24 ± 3.04, 32 ± 5.13, and 18 ± 3.35%, respectively (Figure 3A).

**Figure 3:**
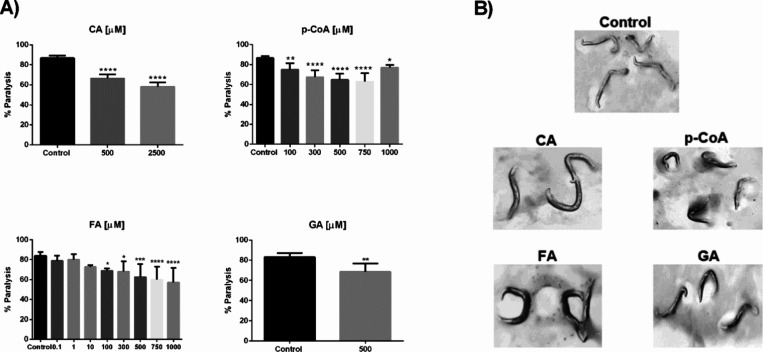
Effect of caffeic (CA), p-coumaric (p-CoA), ferulic (FA), and gallic (GA) acids on β-amyloid aggregation-induced paralysis in *C. elegans* **.** CL4176 worms were cultured at 25 °C under different concentrations (μM) of phenolic compounds and control. (A) Percent of CL4176 worms paralyzed after 36 h at 25 °C. The graphs show the mean paralysis percentage of four independent experiments with error bars representing SD. Statistical significance was determined by one-way ANOVA test and Sidak’s multiple comparisons * *p* < 0.05, ** *p* < 0.01, *** *p* < 0.001, ****p < 0.0001. (B) Representative photographs of the paralysis phenotype in CL4176 worms, with or without treatment, after being incubated for 36 h at 25 °C.

These results coincide with the works previously reported by Li et al.^15^ and Wang et al.^13^ using lower concentrations of CA and FA, respectively. However, we have shown that increasing the concentrations of CA and FA also increases the percentage of protection against paralysis derived from the accumulation of β-amyloid.

PolyQ is a peptide consisting of a sequence of several glutamine units, and its aggregation is related to the development of NDs such as HD. To analyze the effect of phenolic acids on polyQ aggregation, we used strain AM141 (*rmIs133 [unc-54p::Q40::YFP]*). These nematodes grow normally at 20 °C, but a temperature shift to 25 °C leads to PolyQ peptide accumulation in the muscle, causing complete paralysis after 8 days. Treatments with 2500 μM CA, 500 μM p-CoA, 500 μM FA, and 500 μM GA induce a reduction in paralysis percentage by 25 ± 1.6, 21 ± 0.61, 26 ± 0.87, and 47 ± 4.95%, respectively. Lower concentrations showed a minor but significant effect (Figure 4). The PolyQ aggregates associated with this paralysis phenotype can be observed under fluorescence microscopy because they are tagged to the yellow fluorescent protein (YFP). Figure 5 shows that 2500 μM CA, 500–1000 μM p-CoA, 10–1000 μM FA, and 500 μM GA significantly (*p* < 0.01 and *p* < 0.0001) decrease the number of aggregates in worms compared to the control. The same concentrations, except for p-CoA, reduced the percentage of paralysis.

**Figure 4:**
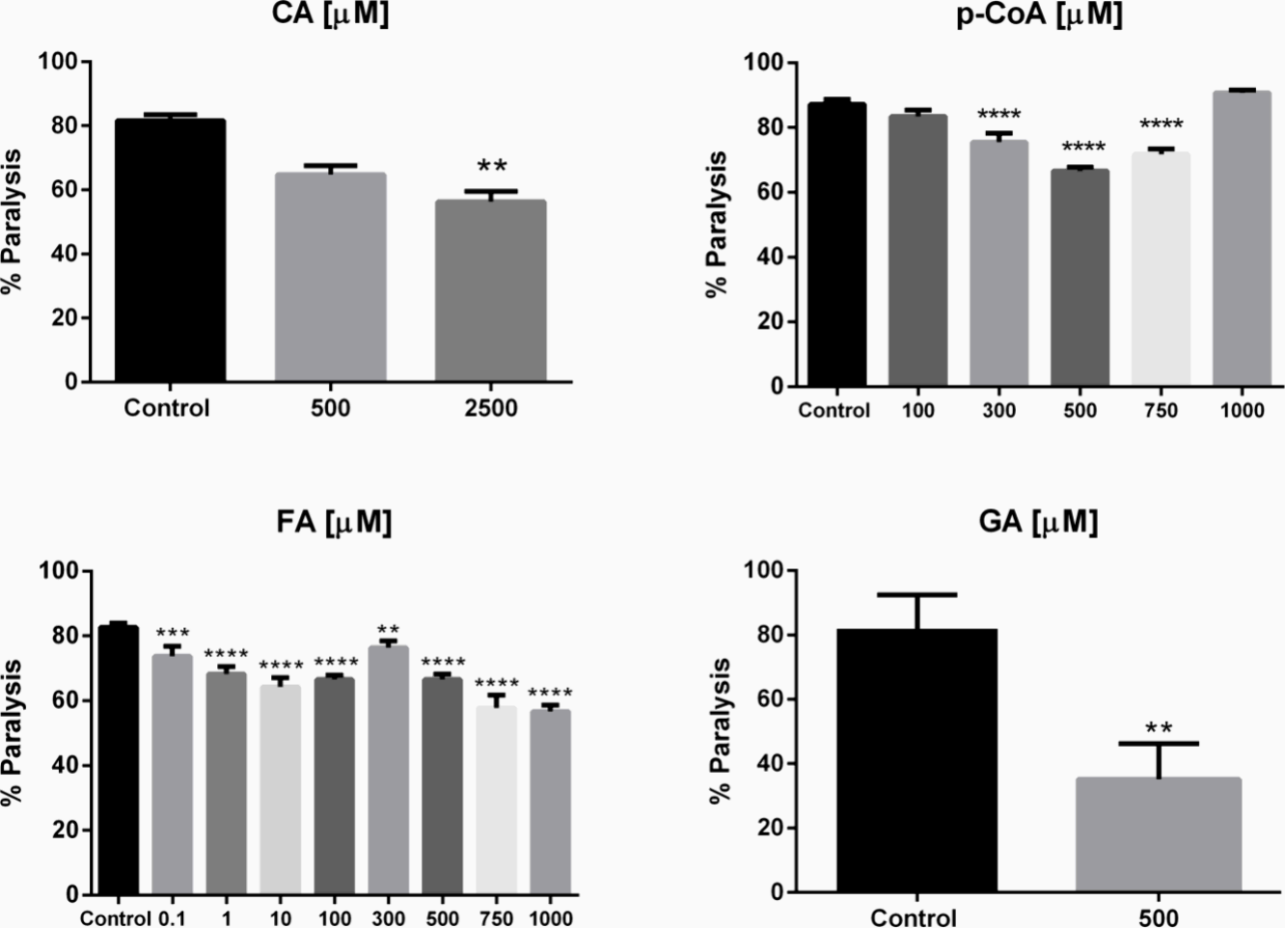
Effect of caffeic (CA), p-coumaric (p-CoA), ferulic (FA), and gallic (GA) acids on polyQ aggregation-induced paralysis in *C. elegans* **.** AM141 worms were cultured at 25 °C under different concentrations (μM) of phenolic compounds and control. Graphs show the mean percent of worms paralyzed after 8 days at 25 °C in four independent experiments with SD error bars. Statistical significance was determined by the one-way ANOVA test and Dunnet’s multiple comparisons ** *p* < 0.01, *** *p* < 0.001, ****p < 0.0001.

**Figure 5:**
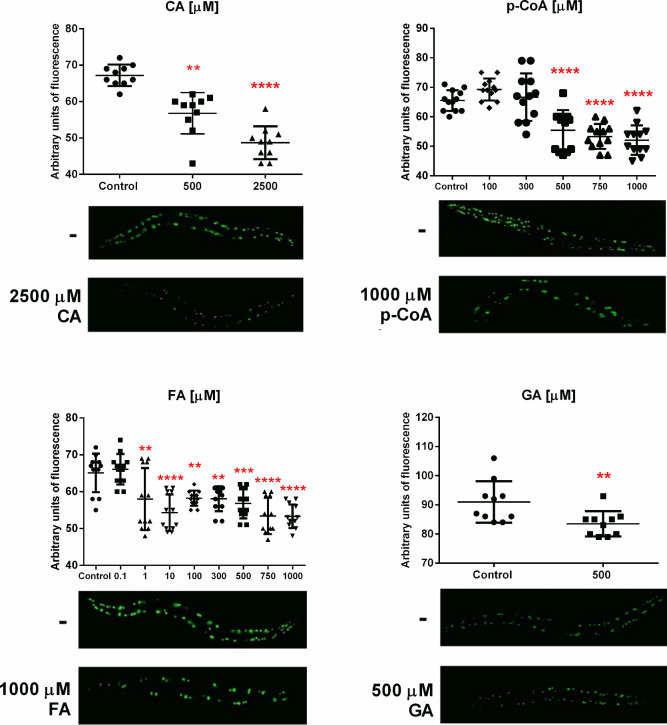
Effect of caffeic (CA), p-coumaric (p-CoA), ferulic (FA), and gallic (GA) acids on polyQ aggregation in *C. elegans* **.** AM141 worms were cultured at 25 °C under different concentrations (μM) of phenolic compounds and control. Graphs show quantification of the aggregate fluorescence in the whole body of ten AM141 worms after 8 days at 25 °C. Statistical significance was determined by one-way ANOVA test and Dunnet’s multiple comparisons ** *p* < 0.01, *** *p* < 0.001, ***p < 0.0001. Below each graph are the representative fluorescence microscope images (20×) of AM141 worms after 7 days of incubation at 25 °C, with control (top) or compound with their respective concentration that showed the least number of aggregates (bottom).

The reduction of polyQ aggregates induced by CA is in line with the work of Bak et al., showing that CA derivate, caffeic acid phenethyl ester treatment can reduce behavioral deficits in a chemical model of Huntington disease (male C57BL/6 mice) and also reduce the mortality of cultures striatal neurons of the same experimental model.^28^

Interestingly, we found that concentrations ranging from 300 to 750 μM p-CoA significantly reduced the percentage of paralyzed worms. However, the 300 μM concentration does not result in a significant reduction in polyQ aggregate production. Higher concentrations (500–1000 μM) of p-CoA are required to reduce the formation of these aggregates; however, the 1000 μM concentration does not improve the paralysis phenotype. These results suggest that p-CoA induces a hormetic response. Hormesis may be defined as a biphasic dose/concentration response displaying a low-dose stimulation and a high-dose inhibition or vice versa.^29^ This could be explained by the fact that each concentration affects different molecular mechanisms. While a high concentration of p-CoA could activate genes associated with autophagy and mitochondrial metabolic stress, low concentrations could inhibit genes that promote polyQ aggregation. The hormetic effect of phenolic acids as neuroprotectors had been previously reported in works that analyzed FA in different biological models^30^; however, this work provides the first assessment of the capacity of p-CoA to induce hormetic dose responses in a biological system.

Finally, we explored the neuroprotective effect of phenolic acids on PD using the model of *C. elegans* that overexpresses α-synuclein fused to a fluorescent reporter in the body wall muscles, the NL5901 (*pkls2386 [unc-54p::*α*synuclein::YFP + unc-119 (+)]*) strain.^31^ The constitutive expression of α-synuclein fused to YFP enables visualization of the accumulation of α-synuclein aggregates throughout the worm’s muscles.

The presence of aggregates in the whole worm was confirmed, and we focused on the α-synuclein aggregates around the pharynx, where aggregates are easily visualized and could be used to compare between control and treatments. After 7 days of treatment at 25 °C, significantly (*p* < 0.01 and *p* < 0.0001) fewer arbitrary fluorescence units (afu) were observed in this area of worms treated with 2500 μM CA (1.63 ± 0.79 afu), 100 μM FA (2.87 ± 0.96 afu), and 500 μM GA (2.49 ± 0.84 afu) compared to the control group (3.67 ± 1.28 afu) (Figure 6). However, treatment with 750 μM p-CoA results in a small but significant (*p* < 0.05) increase (3.8 ± 0.29 afu). Higher and lower concentrations of the treatments did not produce significant changes in the fluorescence.

**Figure 6:**
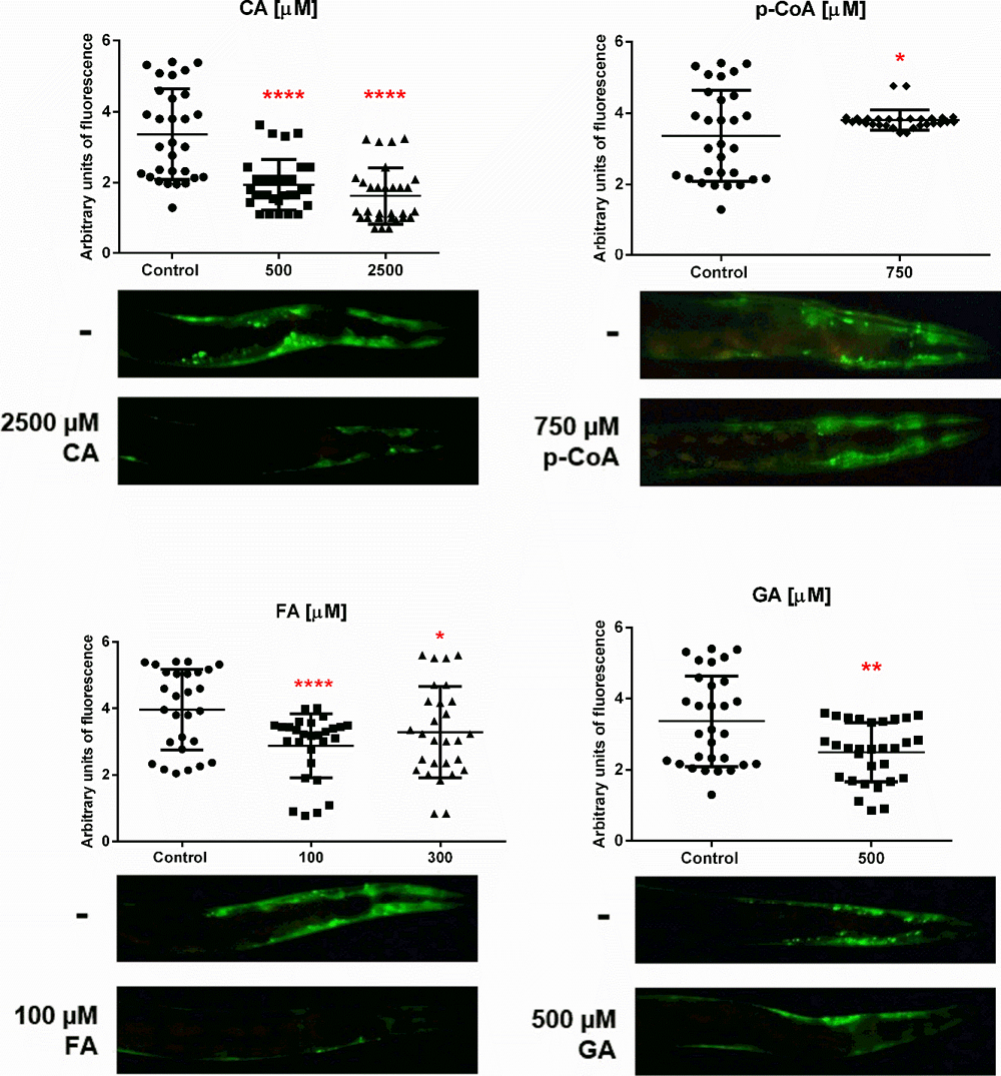
Effect of caffeic (CA), ferulic (FA), and gallic (GA) acids on α-synuclein aggregation in *C. elegans* **.** NL5901 worms were cultured at 25 °C under different concentrations (μM) of phenolic compounds and control. Graphs show quantification of the aggregate fluorescence in the head of 30 NL5901 worms after 7 days at 25 °C. Statistical significance was determined by the one-way ANOVA test and Dunnet’s multiple comparisons * *p* < 0.05, ** *p* < 0.01, ****p < 0.0001. Below each graph are the representative fluorescence microscope images (60X) of NL5901 worms after 7 days of incubation at 25 °C, with control (top) or compound with their respective concentration that showed the least number of arbitrary units of fluorescence (bottom).

As described above, our results show a decrease in α-synuclein aggregation around the pharynx when the nematodes were treated with high concentrations of CA, p-CoA, and lower concentrations of FA, in line with the results found by Long et al. using 100 μM FA.^32^ Additionally, our results demonstrate that GA induces a small but significant (*p* < 0.01) increase in α-synuclein aggregation compared to the control, which contradicts studies on A53TαS from transgenic mice^33,34^ and human neuroblastoma cells in vitro.^34^ Moreover, α-synuclein aggregation is not the only protein involved in PD. A recent study has examined the effects of GA in a rat model of PD induced by rotenone, and it shows that GA improves symptoms of PD in terms of motor, cognitive, intestinal transit time, and thalamic nuclei electrical activity.^35^ Therefore, more studies of the GA effect in PD are required.

## Conclusions

We found that different concentrations of phenolic acids, CA, p-CoA, FA, and GA, can decrease the paralysis phenotype elicited by the aggregation of proteins commonly associated with neurodegeneration in different genetic models generated in *C. elegans*. We observed a significant neuroprotective effect across all three models evaluated by testing a diverse range of phenolic acid concentrations. The protection level depends on the concentration of each compound and the genetic model being tested. However, it is clear that CA and FA provide better protection than p-CoA and GA in the three models, which is probably related to the chemical structure of these molecules, since CA and FA are structurally related. It is also possible that the polarities of P-CoA and GA decrease the bioavailability of these two compounds. Our results suggest that it might be worth testing natural compounds in a wide range of concentrations, since concentrations that are too high or too low may show no effect and lead to the wrong conclusion regarding the potential neuroprotective effect of a particular compound. Additionally, the neuroprotective properties of these compounds can be linked to the beneficial effects observed on foods that contain them, like berries,^36^ nuts,^37^ coffee,^38^ beer,^39^ and whole grains.^40^

In summary, our data establish a significant and positive role for phenolic compounds in reducing protein aggregation associated with the development of NDs. We demonstrated that CA, p-CoA, FA, and GA ameliorate β-amyloid, polyQ, and α-synuclein aggregation. Therefore, these compounds should be considered candidates for translational research because they could have a beneficial impact on human health. In particular, CA and FA are excellent candidates to be neuroprotectors, particularly for diseases related to β-amyloid, polyQ, and α-synuclein aggregation. Although *C. elegans* is a simple and short-lived organism, it has been determinant in discovering the molecular mechanisms underlying aging, and it has been used as a suitable animal model to uncover novel compounds targeting aging pathways. We hope that studies similar to the one that we are presenting now can contribute to uncovering new potential neuroprotectors.

## Methods

### Chemicals and Strains

The *C. elegans* strains N2 (wild-type strain), CL4176 *(smg-1ts [myo-3/A*β*1–42 long 3′-UTR])*, AM141 *(rmIs133 [unc-54p::Q40::YFP])*, NL5901 *(pkls2386 [unc-54p::*α*synuclein::YFP + unc-119 (+)]),* and OP50 bacteria to feed the nematodes were provided by the *Caenorhabditis* Genetic Center (CGC).

Caffeic acid (CAS: 331–39–5), p-coumaric acid (CAS: 501–98–4), ferulic acid (CAS: 537–98–4), gallic acid (CAS: 149–91–7), 5-fluoro-2-deoxyuridine (FUdR, CAS: 50–91–9), levamisole hydrochloride (CAS: 16595–80–5), and all the compounds required to prepare the nematode growth media (NGM) were purchased from SIGMA-Aldrich (MA, USA).

### *C. elegans* Maintenance and Treatment

The nematodes were grown in 6 mL NGM agar plates at 20 °C and fed with concentrated heat-killed (65 °C for 30 min) OP50 bacteria. All the phenolic acids were diluted in 50% ethanol. 100 μL of CA, p-CoA, FA, or GA were added to plates of NGM to achieve the final concentration, and controls were added with 100 μL of 50% ethanol. For the experiments, 37.5 μM FUdR was added to prevent progeny. To ensure that the worms are at the same developmental stage in every experiment, 30 egg-layers were incubated in 60 mm plates at 20 °C. All adult nematodes were removed after 4–5 h, and the synchronized worms were used for each experiment.

### Thermotolerance Assays

Thirty synchronized N2 worms at day 1 of adulthood were exposed to CA, p-CoA, FA, GA (ranging from 0.001 to 2500 μM) or vehicle for 48 h at 20 °C. After pretreatment, the temperature was shifted to 35 °C and, after 8 h, worms were monitored as live or dead every 2 h until the last animal died. Worms were considered dead when they did not respond to a gentle touch and showed no pharyngeal pumping. At least six biological replicates were performed in duplicate for each experiment.

### β-Amyloid Aggregation-Induced Paralysis

Thirty CL4176 (*smg-1ts [myo-3/A*β*1–42 long 3′-UTR]*) synchronized nematodes were treated as described above with different concentrations of CA (500 and 2500 μM), p-CoA (100, 300, 500, 750, and 1000 μM), FA (0.1, 1, 10, 100, 300, 500, 750, and 1000 μM), and GA (500 μM). Young adult CL4176 nematodes were shifted to 25 °C in the presence or absence of these phenolic acids. After 36 h, aggregation-induced paralysis was monitored. Worms were considered paralyzed when they only moved the head in response to a gentle touch, and a *halo* of digested bacteria was observed around the head.

### PolyQ Aggregation-Induced Paralysis

Thirty AM141 (*rmIs133 [unc-54p::Q40::YFP]*) synchronized nematodes were pretreated with CA (500 and 2500 μM), p-CoA (100, 300, 500, 750, and 1000 μM), FA (0.1, 1, 10, 100, 300, 500, 750, and 1000 μM), and GA (500 μM) at 20 °C for 48 h, and then shifted 25 °C to induce protein aggregation. After 8 days, aggregation-induced paralysis was monitored. After 7 days of treatment, AM141 nematodes were mounted on 1% agarose pads and paralyzed with 100 μM levamisole to quantify poly Q aggregation. Images of 10 worms per condition were captured at 20× magnification with an AMSCOPE fluorescence microscope using a GFP filter. The aggregates of whole animals were counted and compared between treatments using the open-source Fiji/ImageJ software (NIH, USA).

### α-Synuclein Aggregation-Induced Paralysis

Thirty NL5901 *(pkls2386 [unc-54p::*α*synuclein::YFP + unc-119 (+)])*synchronized nematodes were treated with CA (500 and 2500 μM), p-CoA (100, 300, 500, 750, and 1000 μM), FA (0.1, 1, 10, 100, 300, 500, 750, and 1000 μM), and GA (500 μM) at 25 °C. After 7 days of treatment, AM141 nematodes were mounted on 1% agarose pads and paralyzed with 100 μM levamisole. Images of 30 worms per condition were captured at 60× magnification with an AMSCOPE fluorescence microscope using a GFP filter. Images were analyzed, and fluorescence was quantified using the open-source Fiji/ImageJ software (NIH, USA).

### Statistical Analysis

All of the experiments in this study were replicated 4–6 times. Kaplan–Meier survival curves were analyzed using GraphPad Prism 8 software (CA, USA). Statistical significance among groups was calculated using the Kruskal–Wallis test or one-way ANOVA test and a post hoc multiple comparison analysis, as indicated in each figure.

## Abbreviations

AD: Alzheimer’s disease
CA: caffeic acid
FA: ferulic acid
GA: gallic acid
HD: Huntington’s disease
ND: neurodegenerative disease
p-CoA: p-Coumaric acid
PD: Parkinson’s disease
YFP: yellow fluorescent protein
